# Vitamin K2 protects mice against non-alcoholic fatty liver disease induced by high-fat diet

**DOI:** 10.1038/s41598-024-53644-6

**Published:** 2024-02-06

**Authors:** Peizuo Zhao, Weidong Yang, Huiyu Xiao, Shuaishuai Zhang, Chuanzhou Gao, Hua Piao, Lihong Liu, Shuzhuang Li

**Affiliations:** 1 Department of Physiology, Dalian Medical University, Dalian, Liaoning People’s Republic of China; 2 Central Laboratory, Dalian Medical University, Dalian, Liaoning People’s Republic of China

**Keywords:** Fat metabolism, Homeostasis, Metabolic diseases, Mitochondria, Diabetes, Obesity

## Abstract

Non-alcoholic fatty liver disease (NAFLD) is one of the most common liver diseases worldwide and there is a huge unmet need to find safer and more effective drugs. Vitamin K has been found to regulate lipid metabolism in the liver. However, the effects of vitamin K2 on NAFLD is unclear. This study aims to evaluate the preventive and therapeutic effects of vitamin K2 in the process of fatty liver formation and to explore molecular mechanisms the associated with lipid metabolism. A non-alcoholic fatty liver model was established by high-fat diet administration for three months. Vitamin K2 significantly reduced the body weight, abdominal circumference and body fat percentage of NAFLD mice. Vitamin K2 also showed histological benefits in reducing hepatic steatosis. NAFLD mice induced by high-fat diet showed increased HMGR while vitamin K2 intervention could reverse the pathological lterations. Adiponectin (APN) is an endogenous bioactive polypeptide or protein secreted by adipocytes. We detected APN, SOD, AlaDH and other indicators that may affect the state of high-fat diet mice, but the experimental results showed that the above indicators did not change significantly. It is worth noting that the effect of vitamin K2 supplementation on the lipid-lowering effect of uc OC in vivo needs to be further explored. This study first reported the protective effect of vitamin K2 on high-fat diet-induced NAFLD in mice. The protective effect of vitamin K2 may be related to the improvement of lipid metabolism disorder in NAFLD.

## Introduction

In recent years, non-alcoholic fatty liver disease (NAFLD) has become a common hidden health killer of adults and children/adolescents worldwide^1^. European Association for the Study of the Liver defines NAFLD as histologically steatosis  > 5% in hepatocytes or proton density fat fraction  > 5.6% under magnetic resonance imaging, excluding other causes of liver steatosis and setting an alcohol consumption limit (≤ 30 g per day for men and ≤ 30 g per day for women)^2^. According to the histological characteristics of the liver, NAFLD can be divided into two pathological types: non-alcoholic fatty liver (NAFL) and non-alcoholic steatohepatitis (NASH)^3^. NAFL is characterized by liver steatosis and accompanied by slight lobular inflammatory infiltration or not^3^. NASH is characterized by ballooning degeneration of hepatocyte with infiltrated lobular inflammation and fibrosis^3^. There are many factors associated with the onset of NAFLD, which is considered to be a component of the metabolic syndrome by the main stream view^4^. The pathogenesis is mainly related to obesity and insulin resistance. As a result, most patients are obese and susceptible to type 2 diabetes, hypertension or dyslipidemia^3^. However, it has been reported that NAFLD may be secondary to pancreatoduodenectomy in patients without insulin resistance or obesity^5^. In clinical practice, there is no recognized effective way to reverse NAFLD other than liver transplantation. Life interventions, such as weight loss and exercise gain, are the mainstream treatment for NAFLD at present^6^. Recent studies have shown that pioglitazone combined with vitamin E improves liver histological inflammation and ballooning in people with type 2 diabetes and NASH^7^. The search for effective prevention or treatment of NAFLD has become the focus of clinical attention.

Vitamin K is present in the body in two forms: phylloquinone (vitamin K1, PK) and menaquinones (vitamin K2, MKs)^8^. Vitamin K2 (VK2) belongs to the vitamin K family, including many subtypes, which is due to the different side chain lengths composed of isoprene groups (MK-n)^9^. Mk-7 has an extremely long half-life in the body, up to 68 hours^10^. By contrast, PK has a half-life of only 1–2 hours^10^. Therefore, MK-7 is relatively stable in the cycle and is also the main form of MK in the body^11^. In recent years, it has been increasingly recognized that adequate vitamin K2 supplies play an important role in many disease states. For example, vitamin K2 fights vascular calcification^12^, osteoarthritis^13^ and asthma^14^. In recent years, more attention has been paid to the surprising role of vitamin K2 in regulating the progression of endocrine disorders. MK-7 significantly improved body fat mass, insulin resistance and serum triglyceride levels in patients with polycystic ovary syndrome^15^. In addition, consistent intake of MK-7 (360 µg/day) can reduce fasting blood glucose and glycated hemoglobin levels in patients with type 2 diabetes^16^. Notably, a case–control study of 348 subjects from Korea showed that adequate vitamin K intake was beneficial in reducing the risk of NAFLD^17^. The previous studies of Vitamin K2 mainly focused on enhancement of liver regeneration and inhibition of hepatocellular carcinoma cells^18,19^, whether vitamin K2 has a preventive and protective effect on NAFLD and the relevant mechanism remain to be further studied.

The purpose of this study was to investigate the preventive and therapeutic effects of vitamin K2 on the formation of NAFLD in mice, and to study the mechanism of vitamin K2 affecting NAFLD.

## Materials and method

### Chemicals and reagents

Vitamin K2 was provided by Sungen Bioscience Co., Ltd (Shantou, China). The purity of the drug was 99.63%. Biochemical kits total cholesterol (TC), high-density lipoprotein (HDL), alaninea minotransferase (ALT), aspartate aminotransferase (AST) and superoxide dismutase (SOD) were from Nanjing Jiancheng Bioengineering Institute (Nanjing, China). ELISA kit adiponectin (APN) was purchased from Shanghai Langton Biotechnology Co., Ltd (Shanghai, China). Three-hydroxy-3-methyl-glutaryl-coenzyme A reductase (HMGR), uncarboxylated osteocalcin (ucOC) and alanine dehydrogenase (AlaDH) were purchased from Jiangsu Enzyme Labeled Biotechnology Co., Ltd (Yancheng, China).

### Experimental design

C57BL/6 male mice were fed in an animal house with a constant temperature of 23 ± 2 °C, humidity of 60–75% and alternating light and dark per 12 h. Based on our previous studies^20^ and preliminary experiments, we determined the dose of VK2 intervention in this experiment. After one week of adaptive feeding, a total of 42 mice were randomly divided into seven groups (n = 6): control group (C), control + vitamin K2 (0.1 mg/kg, 0.2 mg/kg and 0.4 mg/kg) group (C + VK2), high fat diet group (HFD), high fat diet + 0.1 mg/kg vitamin K2 group [HFD + VK2(L)], high fat diet + 0.2 mg/kg vitamin K2 group [HFD + VK2(M)], high fat diet + 0.4 mg/kg vitamin K2 [HFD + VK2(H)] group and high fat diet + vehicle group (HFD + V).

### Animals and models

C57BL/6 male mice (aged 6–8 weeks, 20 ± 2 g) were purchased from SPF Experimental Animal Center of Dalian Medical University.

All control mice were given standard chow diet and all NAFLD mice were given a high-fat diet. High-fat diet contained 26.2 g protein, 26.3 g carbohydrates and 34.9 g fat per 100 g (Jiangsu Xietong Pharmaceutical Bio-engineering Co., Ltd.). Vitamin K2 in different doses group was given daily along with diet for 8 weeks. Vitamin K2 was dissolved in soybean oil and administered by gavage. The criteria for successful modeling were body weight increase by 20% and liver vacuolization under HE staining.

During the modeling of NAFLD, the changes of diet intake and abdominal circumference were recorded every three days, and the body weight of the mice was weighed once a week. A small animal body composition analyzer was used every four weeks to analyze the body composition of mice, including body fat, body fluid and lean body mass weight. The ratio of body fat to body weight is defined as body fat percentage. On the last day of modeling, the mice were euthanized with pentobarbital after fasting for 12 h, and blood and tissue samples were harvested immediately. The liver appearance, size, color change and visceral fat distribution of each mouse were observed and recorded. Similarly, the wet weight of liver and the fat weight around epididymis were measured.

### Ethics statement

This article’s animal experiments were conducted according to the Guide for the Care and Use of Laboratory Animals and were authorized by the Ethics Committee of Dalian Medical University (AEE20051). All animal experiments were implemented in accordance with the recommendations outlined in the ARRIVE guidelines. All methods were performed in accordance with the relevant guidelines and regulations.

### Serum analysis

Serum concentrations of TC, HDL and SOD were measured using their biochemical test kit. Levels of APN, HMGR, ucOC, and AlaDH were measured using an Elisa kit as mentioned above. The plasma samples are stored at  − 80℃ until analysis.

### Histological analysis

Several pieces of liver tissue were taken from the left lobe of the liver. Some of the liver tissues were placed in 10% formalin solution and then paraffin embedding, sectioning and HE staining were performed to observe liver steatosis, inflammation and microgranuloma, liver cell vacuolation and liver fibrosis. Fatty liver staining scores were performed on the liver tissue. Specifically, three fields were randomly selected for each section to observe the hepatic steatosis and the degree of steatosis (steatosis cell number/total cell number) was scored from 0 to 4. (0: no significant degeneration; 1: ≤ 25%; 2: 25–50%; 3: 50–75%; 4: > 75%).

### Data analysis

All data were presented as means ± SEM and evaluated statistically using GraphPad Prism software 8.1. Statistical comparisons were carried out using one-way analysis of variance (ANOVA) with Tukey’s multiple comparison test. Statistical significance was defined as *p* < 0.05. The results of ELISA were analyzed by Gen5, ELISACalc and other software. The OD values read by the microplate reader were used for standard curve fitting and concentration calculation.

## Results

### Effects of vitamin K2 on basic physiological indexes of mice

The altered basic physiological indexes in mice are the important indicators of NAFLD. The following four aspects were examined.

#### Effects of vitamin K2 on body weight, abdominal circumference, food intake and water intake in NAFLD mice

**Figure 1:**
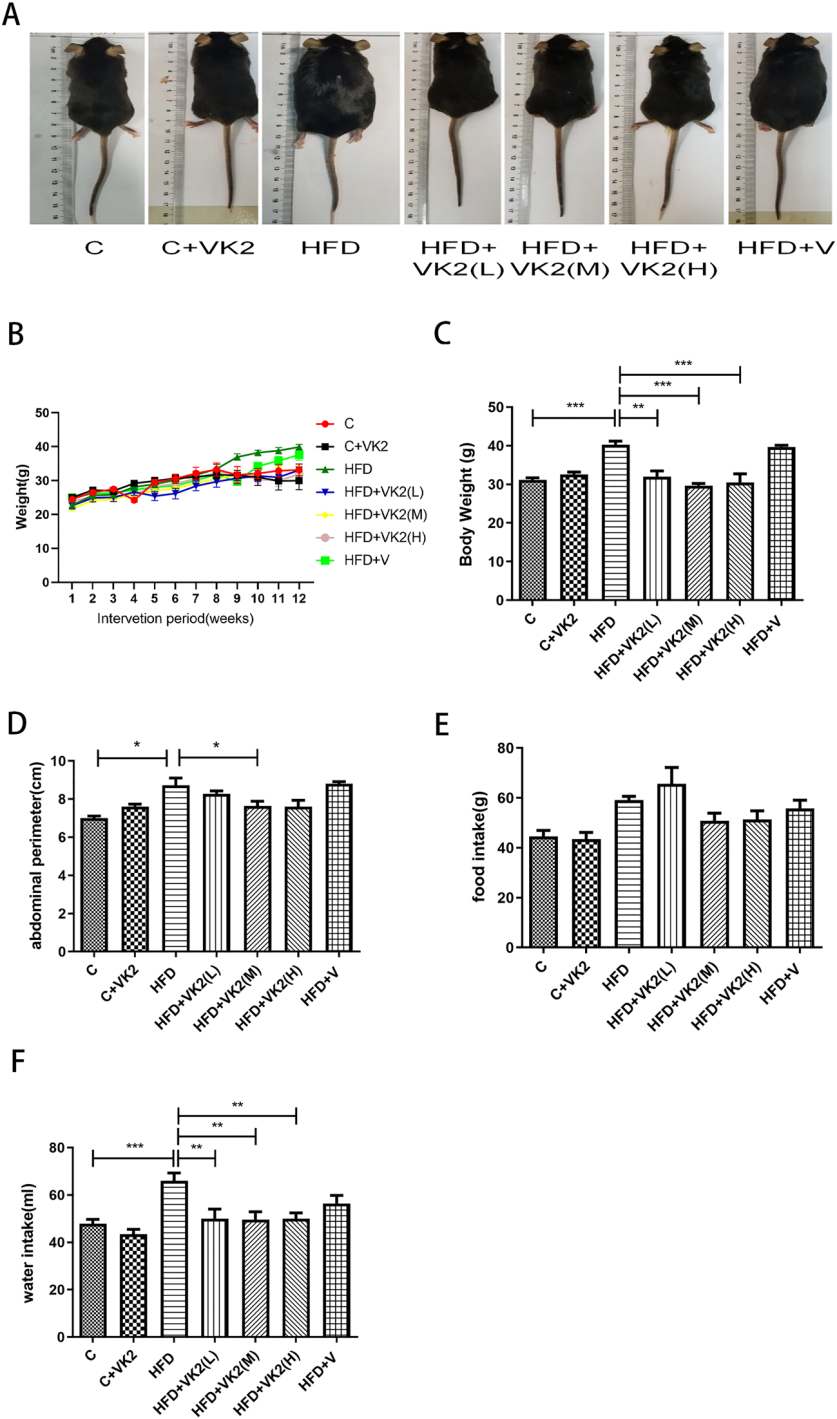
Effects of vitamin K2 on body weight, abdominal circumference, food intake and water intake in mice fed with high fat diet. (**A**) Effects of vitamin K2 on Body size in high-fat diet fed mice for 12 weeks (**B**) Effects of vitamin K2 on body weight changes of each group. (**C**) Effects of vitamin K2 on body weight in 12 weeks. (**D**) Effect of different doses of vitamin K2 on abdominal circumference in high-fat diet fed mice (**E**) Effect of different doses of vitamin K2 on food intake in high-fat diet fed mice. (**F**) Effect of different doses of vitamin K2 on water intake in high-fat diet fed mice. The meaning of abbreviations in the figure: C: control group; C + VK2: control + vitamin K2 group; HFD: high fat diet group; HFD + VK2(L): high fat diet + 0.1 mg/kg vitamin K2 group; HFD + VK2(M): high fat diet + 0.2 mg/kg vitamin K2 group; HFD + VK2(H): high fat diet + 0.4 mg/kg vitamin K2 group; HFD + V: high fat diet + vehicle group. Data are expressed as mean ± SEM (n = 5). *p < 0.05, **p < 0.01; ***p < 0.001.

The representative mice under different interventions were shown in Fig. 1A. HFD mice were significantly larger and obese than control mice, while mice in the vitamin K2 intervention groups were relatively smaller (Fig. 1A).

NAFLD mouse models were successfully induced by feeding mice with high fat diet for 12 weeks. The results of weight change rate in different groups per week (Fig. 1B) revealed that mice gradually gained weight, and there was no significant difference in weight gain among each group in the early stage of the experiment. From week 8, the weight gain trend slowed or even decreased in mice with vitamin K2 intervention compared with HFD group. After 12 weeks, compared with the blank group, the body weight of mice fed with high-fat diet increased significantly, which increased by about 1.3-fold. Compared with the mice in the high-fat diet group, the body weight of the mice in the vitamin K2 intervention group was significantly reduced by about a quarter (Fig. 1C). The changes of abdominal circumference in mice are shown in Fig. 1D. The abdominal circumference of HFD group increased significantly, while the vitamin K2 intervention mice remained relatively stable from week 8. The food intake of mice did not change significantly in each group (Fig. 1E), and the water intake of mice in the HFD group was significantly higher than that in the control group (*p* = 0.0006, Fig. 1F). After vitamin K2 intervention, the amount of drinking water decreased significantly.

#### Effects of vitamin K2 on body composition in week 12

**Figure 2:**
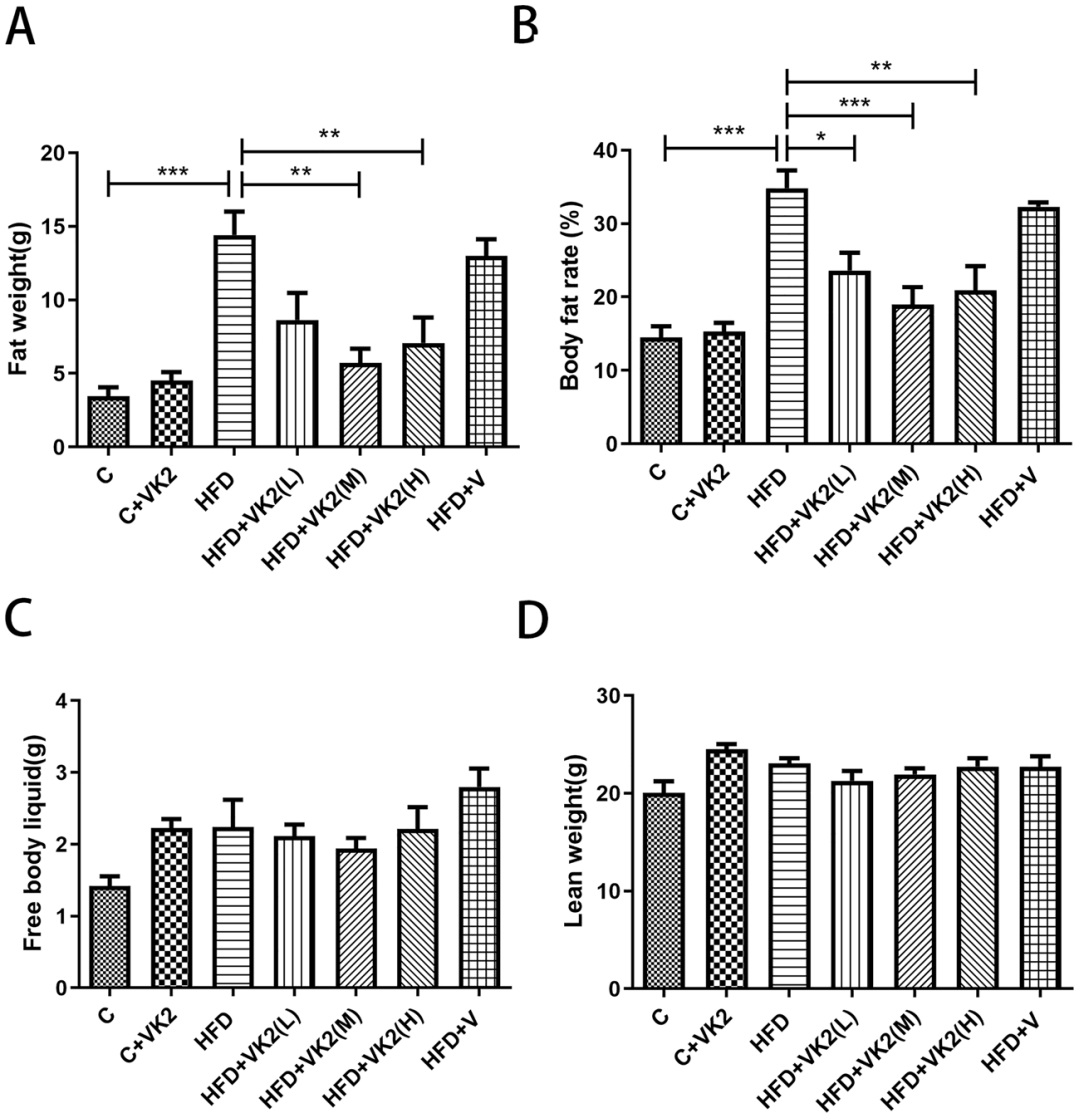
Effects of vitamin K2 on gross appearance and body composition in week 12. (**A**) Body fat weight of mice on a high-fat diet for 12 weeks. (**B**) Body fat rate of mice on a high-fat diet for 12 weeks. (**C**) Free body liquid weight of mice on a high-fat diet for 12 weeks. (**D**) Lean weight of mice on a high-fat diet for 12 weeks. The meaning of abbreviations in the figure: C: control group; C + VK2: control + vitamin K2 group; HFD: high fat diet group; HFD + VK2(L): high fat diet + 0.1 mg/kg vitamin K2 group; HFD + VK2(M): high fat diet + 0.2 mg/kg vitamin K2 group ; HFD + VK2(H): high fat diet + 0.4 mg/kg vitamin K2 group; HFD + V: high fat diet + vehicle group. Data are expressed as mean ± SEM (n = 5).*p < 0.05*;* **p < 0.01, ***p < 0.001*.*

The results of body composition analysis of mice (Fig. 2A) showed that compared with the control group, the body fat weight of mice in the high-fat diet group was significantly increased by about threefold (C vs HFD: *p* = 0.0005). Compared with the high-fat diet group, the body fat weight of the vitamin K2 intervention group was significantly reduced by about twofold (HFD vs HFD + VK2(L): *p* = ns; HFD vs HFD + VK2(M): *p* = 0.0014); HFD vs HFD + VK2(H): *p* = 0.0081). The same trend can also be seen from the statistical chart of body fat percentage (Fig. 2B). Compared with the control group, the body fat rate of the high-fat diet group increased by about 1.7-fold (C vs HFD: *p* = 0.0003); compared with the high-fat diet group, the body fat rate of the vitamin K2 intervention group decreased by about 2.3-fold (HFD vs HFD + VK2(L): *p* = 0.0287; HFD vs HFD + VK2(M): *p* = 0.0010; HFD vs HFD + VK2(H): *p* = 0.0041). However, there were no significant differences in body fluids and lean body mass between the vitamin K2 group and HFD group (Fig. 2C and D). These results suggest that vitamin K2 is effective in reducing fat without affecting the distribution of lean body mass and fluid in the body.

The above results indicate that the mouse model of nonalcoholic fatty liver disease is successfully established and vitamin K2 intervention may have a positive effect on the model.

#### Effects of vitamin K2 on liver

**Figure 3:**
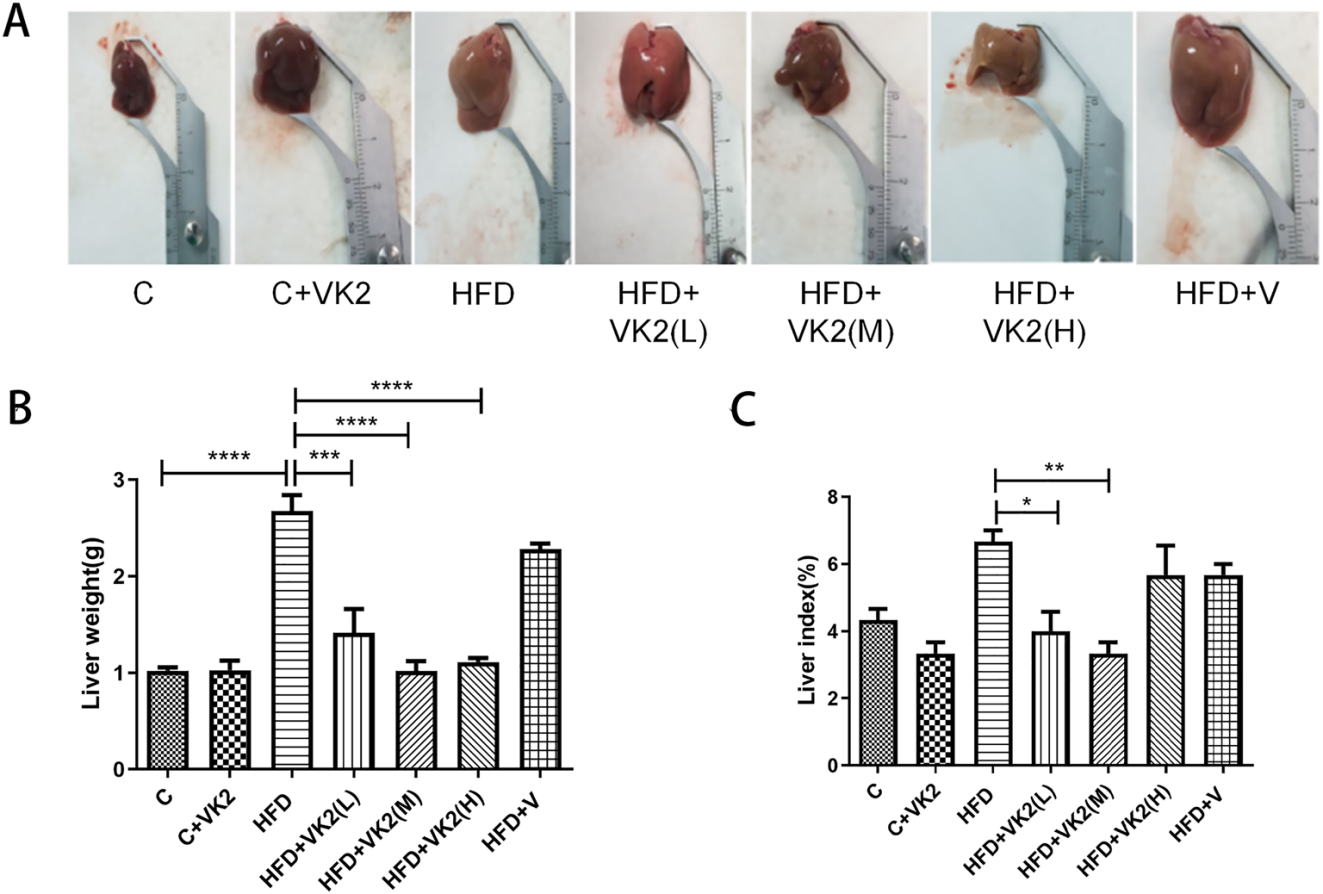
Effect of different doses of vitamin K2 on liver in high-fat diet fed mice. (**A**) Effects of vitamin K2 on Liver specimens in high-fat diet fed mice. (**B**) Effects of vitamin K2 on Liver weight in high-fat diet fed mice. (**C**) Effects of vitamin K2 on Liver index in high-fat diet fed mice. The meaning of abbreviations in the figure: C: control group; C + VK2: control + vitamin K2 group, HFD: high fat diet group; HFD + VK2(L): high fat diet + 0.1 mg/kg vitamin K2 group; HFD + VK2(M): high fat diet + 0.2 mg/kg vitamin K2 group; HFD + VK2(H): high fat diet + 0.4 mg/kg vitamin K2 group; HFD + V: high fat diet + vehicle group. Data are expressed as mean ± SEM (n = 5). *p < 0.05; **p < 0.01; ***p < 0.001; ****p < 0.0001.

The gross view of liver was shown in Supplementary Figure S1. The liver specimens, liver wet weight and liver index (ratio of liver to body weight) were shown in Fig. 3. Visual observation of mouse liver showed that HFD mice livers were significantly larger, smoother and paler than those of control group. However, livers of vitamin K2 intervention mice were relatively ruddy (Fig. 3A, Extended Data Fig. S1). Mice liver weight statistics show that (Fig. 3B), compared with the control group, the liver wet weight of mice in the high-fat diet group was significantly increased by about 2.8-fold (C vs HFD: *p* < 0.0001). Compared with the high-fat diet group, the liver wet weight of the vitamin K2 intervention group was significantly reduced by about 2.7-fold (HFD vs HFD + VK2(L): *p* = 0.0001; HFD vs HFD + VK2(M): *p* < 0.0001; HFD vs HFD + VK2(H): *p* < 0.0001). Similarly, mice liver index (Fig. 3C) analysis showed that, compared with high-fat diet mice, the liver index of vitamin K2 intervention group was significantly decreased by about 1.6-fold [(HFD vs HFD + VK2(L): *p* = 0.0222; HFD vs HFD + VK2(M): *p* = 0.0038]. These results suggest that vitamin K2 can effectively improve the macroscopic damage of the liver. These results suggest that vitamin K2 can effectively improve the macroscopic damage of the liver.

#### Effects of vitamin K2 on fat weight around epididymis

**Figure 4:**
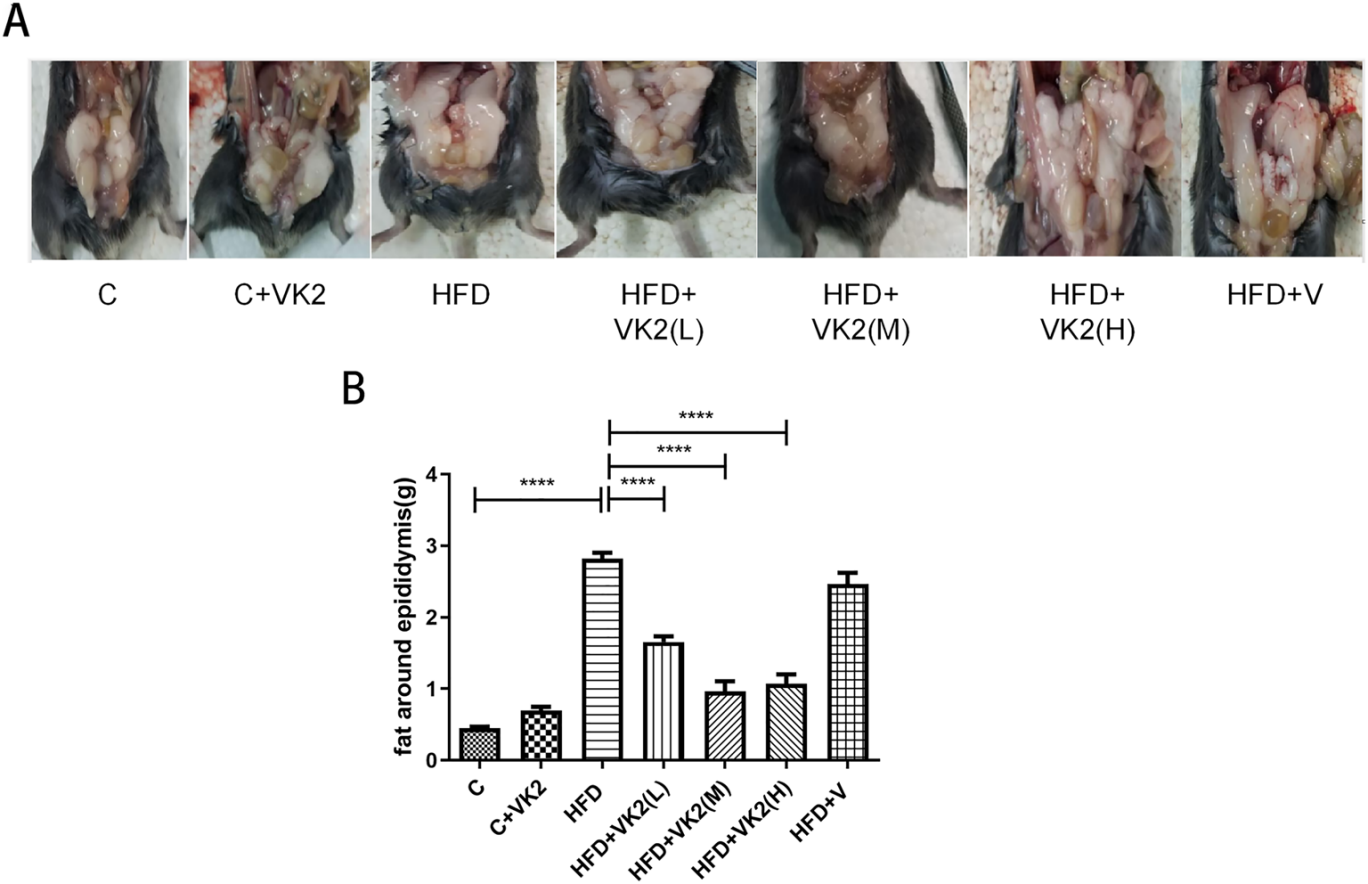
Effect of different doses of vitamin K2 on fat around the epididymis. (**A**) Effects of vitamin K2 on Gross view of fat around the epididymis in high-fat diet fed mice. (**B**) Effects of vitamin K2 on Weight of fat around the epididymis in high-fat diet fed mice. The meaning of abbreviations in the figure: C: control group; C + VK2: control + vitamin K2 group, HFD: high fat diet group; HFD + VK2(L): high fat diet + 0.1 mg/kg vitamin K2 group; HFD + VK2(M): high fat diet + 0.2 mg/kg vitamin K2 group ; HFD + VK2(H): high fat diet + 0.4 mg/kg vitamin K2 group; HFD + V: high fat diet + vehicle group Data are expressed as mean ± SEM (n = 5). ****p < 0.0001*.*

The epididymal fat around the mouse is shown in Fig. 4A. Periepididymal fat reflects visceral fat levels in mice. In the statistics of epididymal fat around mice (Fig. 4B), compared with the control group, the fat accumulation and fat weight around the epididymis in the high-fat diet mice group increased significantly by about sixfold, (C vs HFD: *p* < 0.0001). Compared with high-fat diet mice, vitamin K2 intervention group significantly reduced fat accumulation and fat weight around the epididymis, about 2–3 folds (HFD vs HFD + VK2(L): *p* < 0.0001; HFD vs HFD + VK2(M): *p* < 0.0001; HFD vs HFD + VK2(H): *p* < 0.0001).

These results suggest that vitamin K2 may protect mice against nonalcoholic liver injury by reducing visceral fat burden.

### Effect of vitamin K2 on hepatic histology

**Figure 5:**
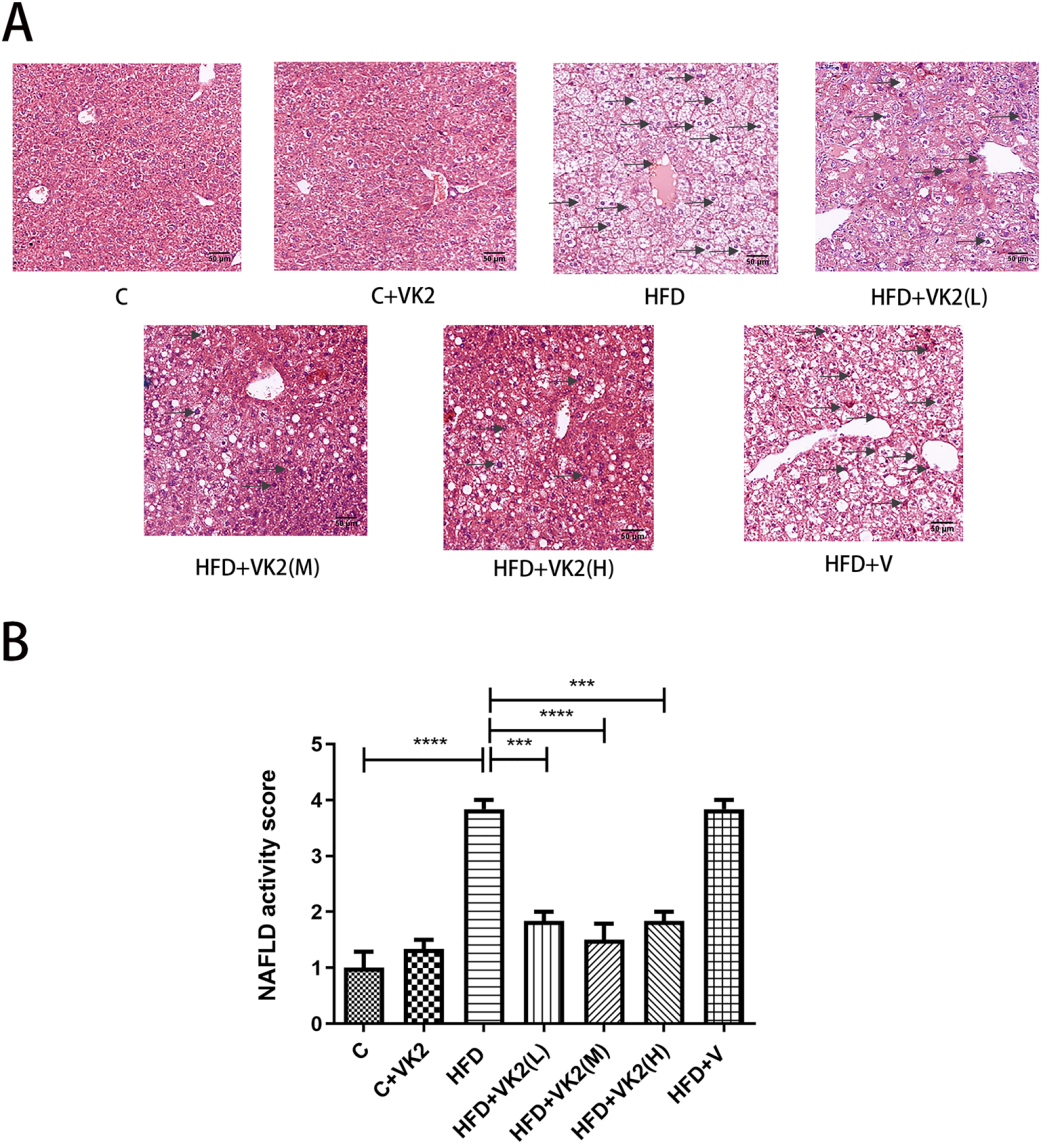
Effect of vitamin K2 on liver histology. (**A**) Liver histological characteristics under light microscope in each group (200X, Scale Bar: 50 μm). The arrows in the figure indicate the location of steatosis, inflammation and fibrosis in the liver. Sections were stained with hematoxylin&eosin. (**B**) Liver fatty degeneration score. The meaning of abbreviations in the figure: C: control group; C + VK2: control + vitamin K2 group, HFD: high fat diet group; HFD + VK2(L): high fat diet + 0.1 mg/kg vitamin K2 group; HFD + VK2(M): high fat diet + 0.2 mg/kg vitamin K2 group; HFD + VK2(H): high fat diet + 0.4 mg/kg vitamin K2 group; HFD + V: high fat diet + vehicle group Data are expressed as mean ± SEM (n = 5). ***p < 0.001*;* ****p < 0.0001*.*

The structures of hepatic cord, hepatic lobule, central vein and portal area in C group and C + VK2 group were clearly visible under light microscope (Fig. 5A). The liver cells were complete and round. The nucleus is large, round, centered with abundant chromatin. Both HFD group and HFD + vehicle group have obviously macrovesicular steatosis which was most obvious in the central lobular portal area and accompanied by hepatocyte ballooning (The color lightens markedly). The hepatic lobule was blurred and the central vein margin was uneven. The nucleus was squeezed to one side and there was inflammation and fibrous hyperplasia. However, the vitamin K2 treatment group saw a significant reduction in hepatic steatosis and the formation of new normal hepatocytes. By analyzing the degree of steatosis in liver tissue (Fig. 5B) of mice in each group : compared with the control group, mice on high-fat diet showed steatosis, which was about fourfold more than that of the control group (C vs HFD: p < 0.0001). Compared with mice on high-fat diet, the degree of steatosis in vitamin K2 intervention group was significantly reduced by twofold (HFD vs HFD + VK2(L): *p* = 0.0001; HFD vs HFD + VK2(M): *p* < 0.0001; HFD vs HFD + VK2(H): *p* = 0.0001).

The results showed that vitamin K2 prevented the pathological changes of liver tissue in mice.

### Effect of vitamin K2 on serological indexes

Based on previous experiments, we have successfully established NAFLD mouse model, and preliminarily validated that vitamin K2 has a positive effect on high-fat diet induced NAFLD, and the optimal dose for NAFLD treatment is 0.2 mg/kg [HFD + VK2(M)].

**Figure 6:**
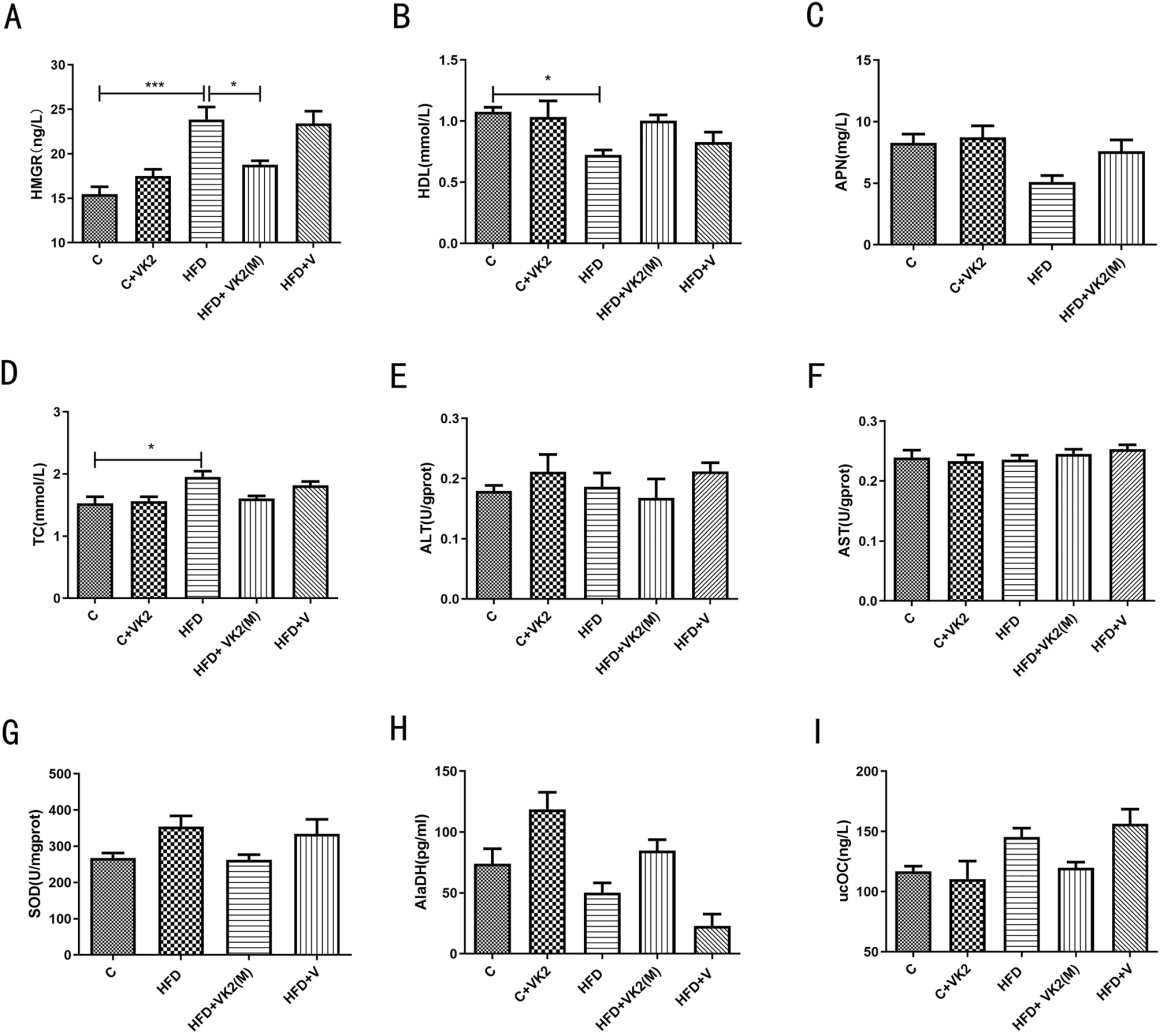
Effect of vitamin K2 on serological indexes, hepatotoxicity and other indexs in high-fat diet fed mice. (**A**) HMGR in serum. (**B**) HDL in serum. (**C**) APN in serum. (**D**) TC in serum. (**E**) ALT in serum. (**F**) AST in serum. (**G**) SOD in high-fat diet fed mice. (**H**) AlaDH in high-fat diet fed mice. (**I**) UcOC in high-fat diet fed mice. The meaning of abbreviations in the figure: C: control group; C + VK2: control + vitamin K2 group, HFD: high fat diet group; HFD + VK2(L): high fat diet + 0.1 mg/kg vitamin K2 group; HFD + VK2(M): high fat diet + 0.2 mg/kg vitamin K2 group; HFD + VK2(H): high fat diet + 0.4 mg/kg vitamin K2 group; HFD + V: high fat diet + vehicle group Data are expressed as mean ± SEM (n = 4). *p < 0.05; *** p < 0.001*.*

Trihydroxy-3-methylglutaryl coenzyme A reductase (HMGR) is a key rate-limiting enzyme in cholesterol synthesis. The results showed that the HMGR level in the high-fat diet group was significantly increased by 1.6-fold compared to control group (C vs HFD: *p* = 0.005). After administration of vitamin K2, the level of HMGR was significantly inhibited by 32% (HFD vs HFD + VK2(M): *p* = 0.0338) (Fig. 6A).

As one major feature of NAFLD is the disorder of lipid metabolism^21^, thus the lipid-related indicators are also specifically detected. TC, and HDL are important clinical indicators of blood lipids^22^. The results showed that TC in the HFD group were significantly (*p* = 0.0141) higher than control group, while HDL was significantly (*p* = 0.0235) reduced. After vitamin K2 intervention, there was no significant change in TC and HDL levels (Fig. 6B and D).

APN is a protein hormone and adipokine produced primarily in adipose tissue that regulates many metabolic processes and predicts early risk of type 2 diabetes^23^. However, in this experiment, APN did not change significantly (Fig. 6C).

The above data indicate that lipid levels and lipid metabolism-related indicators HMGR have changed significantly in the NAFLD mouse model, suggesting that vitamin K2 may improve lipid metabolism disorders.

### Effect of vitamin K2 on hepatotoxicity, oxidative stress and mitochondrial function

Hepatotoxicity may occur on the basis of hepatocyte steatosis. AST and ALT are classic indicators for detecting hepatotoxicity injury^24^. However, the experimental results showed that mice fed with high-fat diet for 3 months did not show significant damage. In addition, vitamin K2 alone or vitamin K2 combined with high-fat diet did not show significant hepatotoxicity (Fig. 6E and F).

Oxidative stress is an important cause of NAFLD disease progression and liver damage. SOD is an antioxidant enzyme that represents the antioxidant level of oxidative stress^25^. However, Fig. 6G showed that there was no significant change in the level of SOD in liver tissue of mice in each group in this experiment.

Mitochondrial homeostasis plays a decisive role in cellular energy metabolism and signal pathway regulation. The imbalance of mitochondrial homeostasis is the key pathological cause of metabolic diseases such as nonalcoholic fatty liver disease and obesity^26^. AlaDH is positively correlated with the function of mitochondrial oxidative respiratory chain^27^. The level of AlaDH in each group were shown in Fig. 6H. However, in this experiment, AlaDH did not change significantly in each group.

### Serum levels of ucOC

Osteocalcin can be involved in the regulation of glycolipid and energy metabolism, which may be related to coronary heart disease and nonalcoholic fatty liver disease^28,29^. Carboxylation is an important process for osteocalcin to function. Fig. 6I shows the serum levels of ucOC in each group. The level of ucOC in the HFD group was higher than control group, suggesting that the low level of vitamin K in the HFD group led to insufficient carboxylation of OC. In this experiment, there was no significant difference in uc OC in each group of mice, so vitamin K2 may not reduce the damage of NAFLD through carboxylated osteocalcin.

## Discussion

The prevalence of NAFLD is as high as 25% of adults worldwide has become an important cause of liver disease all over the world. Therefore, NAFLD deserves great attention from medical workers all over the world.

Our results demonstrate that vitamin K2 has a protective effect on high-fat diet-induced NAFLD in mice. In this study, three different doses of vitamin K2 prophylactic interventions were effective in delaying body weight and abdominal circumference gain in mice (Fig. 1B and D). Dam et al.^30^ conducted a 10-year follow-up study of 802 adults and found that those with high vitamin K2 intake had a lower waist circumference and a lower incidence of metabolic syndrome. In addition, it has been reported that human plasma vitamin K2 level is inversely correlated with body mass index^31^. We found that mice given vitamin K2 showed significant differences in body fat content and body fat percentage (Fig. 2A and B) from NAFLD mice at week 12 respectively. Concretely, vitamin K2 significantly inhibited the increase of body fat and body fat percentage in mice fed high fat diet, and 0.2 mg/kg intervention had the best effect. Notably, vitamin K2 regulates fat metabolism in the body without affecting the consumption or distribution of other components in the body, such as lean body mass and free body fluid (Fig. 2C and D). Our results suggest that vitamin K2 is a safe and effective nutrient for reducing fat and weight in NAFLD with obesity.

Our results showed that NAFLD mice were larger and significantly obese, whereas the body size (Fig. 1A) of the mice was well controlled with vitamin K2 intervention. In addition, we found that prophylactic administration of vitamin K2 not only reduced gross hepatic adipose degeneration, but also effectively reduced visceral fat accumulation (Fig. 4). A randomized controlled trial confirmed our findings that high vitamin K2 intake helped to reduce body weight, abdominal and visceral fat in postmenopausal women.

The protective effect of vitamin K2 on NAFLD was demonstrated in the histopathological examination of liver (Fig. 5). In the NAFLD group, extensive hepatic steatosis was observed under light microscopy. The steatosis was located in the central hepatic lobular portal area, accompanied by inflammatory reaction and fibrous hyperplasia. Salw et al.^32^ explained this phenomenon by showing that liver pathology changes in NAFLD mice induced by a high-fat diet developed gradually over time. However, no obvious inflammatory tendency was found in the detection of AST and ALT in mice (Fig. 6E and F). In other words, in mild NAFLD, fat is stored in the cytoplasm and does not necessarily invade other organelles to cause significant inflammatory changes. Among all the vitamin K2 intervention groups, NAFLD + VK2(M) group had the most obvious changes, liver inflammation was reduced, lipid droplets were small and dispersed, and no significant fibrosis area was observed. Our results suggest that vitamin K2 can decrease liver fat accumulation in mice histologically, and has certain preventive and reversal effects on NAFLD.

In our study, NAFLD mice developed dyslipidemia, which was characterized by increased serum TC levels and decreased HDL levels (Fig. 6D and B). Hepatic TG accumulation is a measure of steatosis, which together with oxidative stress and inflammation may lead to the occurrence of NAFLD^33^. Notably, atherosclerotic dyslipidemia in NAFLD also increases the risk of cardiovascular disease^34^. However, our results showed that vitamin K2 failed to correct dyslipidemia in NAFLD mice. In addition, cholesterol synthesis was significantly upregulated in NAFLD. Studies have shown that the expression of HMGR, as the rate-limiting enzyme of cholesterol synthesis, is significantly increased in both NAFLD and NASH and is independent of whether the subjects are obese or not^35^. Moreover, the high expression of HMGR was closely related to liver free cholesterol and NAFLD activity score^35^. In our study, HMGR levels were significantly reduced with vitamin K2 intervention (Fig. 6A), suggesting that vitamin K2 is involved in ameliorating cholesterol metabolic disorders. APN is one of the most abundant cytokines secreted by adipocytes. APN interacts with the adaptor protein phosphotyrosine through receptors in the liver to further activate multiple signaling pathways^36^. For example, APN activates 5′ adenosine monophosphate-activated protein kinase and peroxisome proliferator-activated receptor-alpha signaling pathways to reduce fatty acid synthesis and promote fatty acid oxidation to inhibit liver fat accumulation^37^. It is worth mentioning that APN can effectively improve serum HDL level and increase insulin sensitivity^37^. Recently, a randomized placebo-controlled trial of 148 postmenopausal women found that long-term MK-7 supplementation significantly increased adiponectin levels^38^. However, our study showed that APN (Fig. 6C) levels in each group of mice did not change significantly compared with other groups, which may indicate that APN is not the focus of our future research.

We also evaluated the liver SOD (Fig. 6G) content although Numerous studies have shown that vitamin K2 is a powerful antioxidant, which can effectively scavenging ROS and improve SOD activity^39,40^. However, our results showed that there was no significant change in SOD content in each group of mice.

In addition, we also found that vitamin K2 prophylactic intervention doesn’t increased alanine dehydrogenase (Fig. 6H) levels in NAFLD mice. Although vitamin K2 has been shown to act as an electron carrier to maintain ATP supply in mitochondria^41^.

Osteocalcin is a well-known member of the vitamin K dependent protein family. After vitamin K carboxylation, osteocalcin is deposited in the mineralized bone matrix, it has been found that uc OC is closely associated with NAFLD, metabolic syndrome, and type 2 diabetes mellitus^42,43^. It has been reported that adult women with serum osteocalcin levels below 25th percentile of whole population have a 90% increased risk of NAFLD^43^. Moreover, osteocalcin is associated with the outcome of NAFLD disease. NAFLD patients with serum osteocalcin levels below 25th percentile of the whole population have a 44% reduced chance of disease remission^43^. Uc OC regulates lipid metabolism in tissues and liver mainly by inducing APN expression in adipocytes, promoting the process of liponysis and triggering adipocyte necrosis^44^. Our results showed that there was no significant change in uc OC in each group of mice (Fig. 6I). Overall, our results suggest that vitamin K2 intervention can improve lipid metabolism disorders in NAFLD, but vitamin K2 supplementation does not seem to have much effect on lipid regulation of uc OC in vivo.

Although the way that vitamin K2 may act on nonalcoholic fatty liver has been explored from many aspects, the final results are relatively few. This is also the limitation of this experiment. Starting from the experiment itself, although the ultimate goal is to explore the molecular mechanism of vitamin K2 acting on non-alcoholic fatty liver, the current experimental results only prove that vitamin K2 is effective for the treatment of NAFLD from the perspective of in vivo. There are many directions for future research, including the most effective dose of vitamin K2 currently obtained is the intermediate dose rather than the maximum dose, which does not conform to the dose dependence. The lack of in vitro experiments is also the lack of this experiment. At present, there are three ways in which vitamin K2 plays a role, namely, by activating mitochondrial function^41^, promoting the carboxylation of vitamin K-dependent proteins^45^, and inhibiting ferroptosis^46^. In future studies, it will be meaningful to explore whether there is a direct relationship between these three methods and known or unknown targets of NAFLD.

## Conclusion

In summary, vitamin K2 has a significant protective effect on high-fat diet-induced NAFLD in mice. Vitamin K2 significantly reduced the fat content of high-fat diet-induced NAFLD mice without affecting the distribution of other body components. Vitamin K2 can also effectively improve the liver histological changes of NAFLD. In addition, vitamin K2 did not improve dyslipidemia, but corrected cholesterol metabolism disorders, which was manifested as a decrease in HMGR compared with the high-fat diet group. In other tests, adiponectin did not change significantly in this test. AlaDH representing mitochondrial function and SOD representing oxidative stress did not show significant differences in each group of this experiment. In this experiment, vitamin K2 intervention did not seem to have much effect on uc OC. However, in general, vitamin K2 supplementation is still helpful to prevent the occurrence of NAFLD and delay the progression of NAFLD. Our study provides a new potential treatment for NAFLD.

### Supplementary Information

Supplementary Information.

## Data Availability

The datasets used and/or analyzed during the current study are available from the corresponding author on reasonable request.
