# Supplementary figures and images for: Vitamin K2 protects mice against non-alcoholic fatty liver disease induced by high-fat diet
Source: Sci Rep. 2024 Feb 6;14:3075. doi: 10.1038/s41598-024-53644-6 (PMC10847165; doi:10.1038/s41598-024-53644-6)

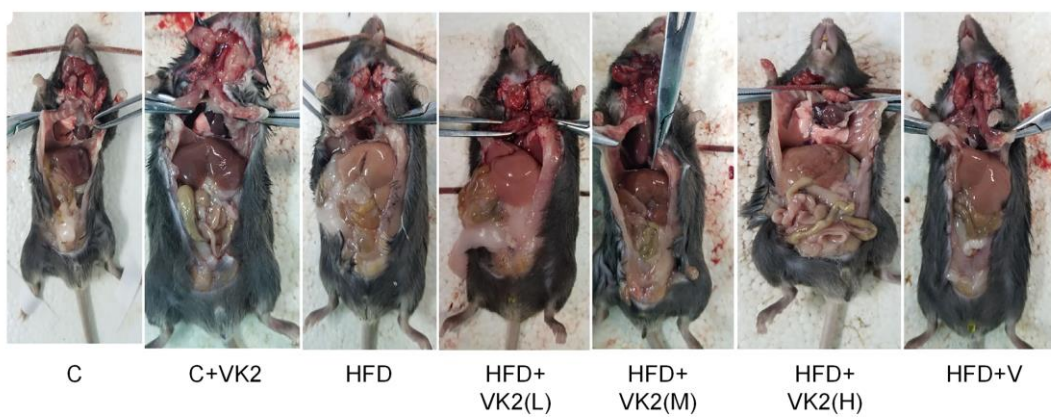

### Supplementary Figure S1

The gross view of liver

Supplement: Supplementary file 1 — Supplementary Information. [file 41598_2024_53644_MOESM1_ESM.pdf]
